# *Mycobacterium tuberculosis* Infection Induces Low-Density Granulocyte Generation by Promoting Neutrophil Extracellular Trap Formation *via* ROS Pathway

**DOI:** 10.3389/fmicb.2019.01468

**Published:** 2019-07-11

**Authors:** Rigu Su, Yi-ping Peng, Zhen Deng, Ya-ting Deng, Jianq-qing Ye, Yang Guo, Zi-kun Huang, Qing Luo, Hong Jiang, Jun-ming Li

**Affiliations:** ^1^Department of Clinical Laboratory, The First Affiliated Hospital of Nanchang University, Nanchang, China; ^2^Department of Tuberculosis, Jiangxi Chest Hospital, Nanchang, China; ^3^Department of Clinical Laboratory, Xiangya Hospital, Central South University, Changsha, China; ^4^Department of Clinical Laboratory, The Second Affiliated Hospital of Nanchang University, Nanchang, China

**Keywords:** *Mycobacterium tuberculosis*, low-density granulocytes, mechanism, neutrophil extracellular traps, reactive oxygen species

## Abstract

The roles and characteristics of low-density granulocytes (LDGs) have recently attracted attention; however, the mechanism of the formation of LDGs is yet unclear. In one of our previous studies, the frequency of LDGs was significantly elevated in the peripheral blood of tuberculosis patients, and *in situ* activation contributed to the generation of LDGs upon *Mycobacterium tuberculosis* infection. However, the underlying molecular mechanisms are yet to be elucidated. In the present study, the release of neutrophil extracellular traps (NETs) and the levels of ROS were regulated before the normal-density granulocytes (NDGs) to be infected with *M. tuberculosis*, and the conversion of NDGs to LDGs was monitored subsequently as well. The results showed that tuberculosis-related LDGs spontaneously released high levels of NETs. Promoting the release of NETs led to increase in the conversion of NDGs to LDGs in *M. tuberculosis* infection, while inhibiting the release of NETs suppressed this conversion after the infection. The *M. tuberculosis* infection significantly increased the ROS levels in neutrophils and the conversion of NDGs to LDGs. Scavenging ROS or blocking the ROS generation of *M. tuberculosis*-infected NDGs significantly suppressed the release of NETs and blocked the generation of LDGs. Moreover, inhibiting the formation of NETs without affecting the levels of ROS significantly decreased the conversion of NDGs to LDGs after *M. tuberculosis* infection. Overall, this study demonstrated that *M. tuberculosis* could induce the generation of LDGs by promoting the release of NET *via* ROS pathway.

## Introduction

Tuberculosis is a chronic infectious disease caused by *Mycobacterium tuberculosis* infection, which is the ninth leading cause of deaths worldwide and the leading cause of deaths from a single infectious agent. Approximately, one-third of the world’s population is estimated to be infected by *M. tuberculosis*. About 10.4 million cases of tuberculosis occur and 1.7 million individuals succumb to death due to this disease annually (World Health Organization, 2017). *M. tuberculosis* does not produce any toxin, exotoxin, or invasive enzymes. The pathological damage by tuberculosis is primarily mediated by the pathological immune response to *M. tuberculosis* infection (Mourik et al., 2017; Scriba et al., 2017). Neutrophils are the first cells that infiltrate in the local tissue after *M. tuberculosis* infection and are the most abundant leukocytes in the airways of patients with pulmonary tuberculosis (Eum et al., 2010). Although some studies suggested that neutrophils might be involved in tuberculosis-related tissue damage and effectuate the activation of latent tuberculosis, however, the specific roles and characteristics of neutrophils in TB infection still need to be elucidated.

Recent studies described a specific subpopulation of neutrophils, termed as low-density granulocytes (LDGs) or low-density neutrophils (LDNs) in the peripheral blood of patients with autoimmune diseases, cancer, and infection (Hacbarth and Kajdacsy-Balla, 1986; Carmona-Rivera and Kaplan, 2013; Sagiv et al., 2015). LDGs are defined as such because these cells are found to sediment in the peripheral blood mononuclear cell (PBMC) fraction after density gradient centrifugation of the peripheral blood (Hacbarth and Kajdacsy-Balla, 1986). Notably, LDGs display different phenotype, maturation status, and function in different studies. Several studies demonstrated that LDGs are activated mature neutrophils that mediate enhanced proinflammatory and cytotoxic responses (Moses and Brandau, 2016). However, some other studies provided evidence to prove that LDGs are immature neutrophils that display immunosuppressive properties (Denny et al., 2010; Grayson et al., 2015).

In a previous study, we found an aberrantly high level of LDGs in the peripheral blood of patients with active tuberculosis, and the frequencies of LDGs were correlated with the severity of tuberculosis. This phenotype suggested the role of these cells in the occurrence of tuberculosis and tissue damage caused by the disease (Deng et al., 2016). Furthermore, we found that the *in vitro* infection with *M. tuberculosis* induces the generation of LDGs in both peripheral blood and purified normal-density granulocytes (NDGs) and that the *in situ* activation contributes to the generation of LDGs in *M. tuberculosis* infection (Deng et al., 2016). However, the underlying molecular mechanisms are still unknown.

Since neutrophils are primarily involved in the tissue damage in tuberculosis, and LDGs are a group of highly activated neutrophils, eliminating the mechanisms that generate the tuberculosis-related LDGs might be crucial for developing the techniques to block the generation of LDGs and promoting the treatment of active tuberculosis. In this study, we reported that the generation of *M. tuberculosis* infection-related LDGs is related to the intracellular ROS level and the release of neutrophil extracellular traps (NETs) in neutrophils.

## Materials and Methods

### Study Subjects

Patients with pulmonary tuberculosis were recruited from the Jiangxi Chest Hospital from April 2017 to July 2017. The diagnostic criteria for tuberculosis were in accordance with the “Guideline of Clinical Diagnosis and treatment: TB section of China” (Chinese Medical Association, 2005). Age- and sex-matched, TST-negative asymptomatic healthy volunteers were recruited from April 2017 to February 2018. All subjects with autoimmune diseases, cancer, current or recent infection were excluded from the study.

This study was approved by the Ethical Review Committees of the First Affiliated Hospital of Nanchang University and was carried out in compliance with the Helsinki Declaration. Written informed consent was obtained from all the subjects enrolled in this study.

### Neutrophils Preparation

Peripheral venous blood was collected in vacuum containers containing EDTA-anticoagulant and processed within 4 h of phlebotomy. PBMCs were isolated by density gradient centrifugation with Ficoll-amidotrizoaat (LUMC, Leiden, Netherlands) as described previously (Deng et al., 2016). Briefly, 3 ml of venous blood was diluted 1:1 with sterile saline and centrifuged on Ficoll for 30 min at 300 ×*g*, 4°C. The PBMCs were collected by aspiration from the plasma-lymphocyte interface. The contaminating RBCs were lysed by erythrocyte lysis (OptiLyse C lysing solution, Beckman Coulter, Brea, CA, USA). The LDGs were purified from PBMC fraction by an immunomagnetic negative selection method using an EasySep Direct Human Neutrophil Isolation Kit (STEMCELL Technologies, Vancouver, BC, Canada) according to the manufacturer’s instructions. The purity of the LDG fraction was typically >95% as determined by staining with CD15-PE-Cy5 and CD14-ECD and analyzing with flow cytometry (FCM). The LDGs were identified as CD14^low^ CD15^+^ granulocytes (Deng et al., 2016). The NDGs were isolated by dextran sedimentation from RBC pellets and erythrocyte lysis. The viability of the cells was monitored by trypan blue staining. Subsequently, the freshly prepared neutrophils were resuspended at a density of 5 × 10^5^ cells/ml in RPMI 1640 medium (Life Technologies, Carlsbad, CA, USA) supplemented with 3% heat-inactivated human AB sera (Sigma-Aldrich, St. Louis, MO, USA).

### Flow Cytometry Analysis

The PBMCs or neutrophils (10^6^ cells each) were washed and incubated with FcR blocking reagent (Milteny Biotec, Auburn, CA, USA) for 5 min, followed by incubation at room temperature for 30 min with or without the mixture of fluorescent-labeled anti-human monoclonal antibodies: CD14-ECD and CD15-PE-Cy5 (Beckman Coulter, Brea, CA, USA). The dead cells were eliminated from the analysis as deduced by staining with Annexin V-FITC and propidium iodide. The cell doublets were eliminated based on the size of the event (FSC). The analysis was performed using a Cytomics FC 500 flow cytometer (Beckman Coulter, Brea, CA, USA), and isotype-matched controls were used to determine auto-fluorescence and nonspecific staining.

### Bacterial Strains and Infection

*M. tuberculosis* H37Rv strain was cultured at 37°C up to early mid-log phase in Middlebrook 7H9 broth, supplemented with 10% OADC growth supplement (BD Bioscience, San Jose, CA, USA) and 0.5% glycerol. To obtain single cell suspension, the bacterial aggregates were diluted in RPMI 1640 medium, mixed with a pipette, and treated with an ultrasonic shaker for 15 min. The solution was left standing for 15 min before the supernatant was collected and density adjusted to 0.5 at OD600 (approximately 10^7^ bacteria/ml). For *in vitro* infection, the purified neutrophils were infected with *M. tuberculosis* at the multiplicity of infection (MOI) of 5 (bacteria to neutrophils) as described previously (Deng et al., 2016). Furthermore, 20 nM phorbol 12-myristate 13-acetate (PMA, Sigma-Aldrich, St. Louis, MO, USA), 10 μM N,N,N,N,-tetrakis-2(pyridyl-methyl) ethylenediamine (TPEN, Sigma-Aldrich), 50 μM hydrogen peroxide (H_2_O_2_, Sigma-Aldrich), 25 μM diphenyleneiodonium (DPI, Sigma-Aldrich), 30 μM N-acetyl-L-cysteine (NAC, Sigma-Aldrich), or 200 μM Cl-amidine (AAT Bioquest, Sunnyvale, CA, USA) was added to the culture for 30 min before infection.

### Determination of Normal-Density Granulocyte-to-Low-Density Granulocyte Conversion

Purified NDGs were pretreated with or without PMA, TPEN, NAC, H_2_O_2_, DPI, or Cl-amidine for 30 min, and then infected with or without *M. tuberculosis* for 2 h at MOI 5. After washing three times with phosphate-buffered saline (PBS), the treated cells were resuspended in 3 ml of sterile saline and added to 3 ml of freshly collected autologous whole blood. Immediately after the treated cells were mixed into the autologous blood, the PBMCs in this mixture and in another equal division of untreated autologous peripheral blood were isolated by Ficoll density gradient centrifugation. The LDGs in these PBMCs were identified as CD14^low^ CD15^+^ cells and enumerated by FCM. The number of LDGs in the treated neutrophil samples was calculated by subtracting the number of LDGs in the untreated autologous blood with LDGs in the mixture.

### Determination of Neutrophil Extracellular Traps

After *in vitro* culture with or without stimulation or infection, the neutrophils were fixed with 4% formaldehyde, permeabilized by 0.1% Triton X-100, and then blocked with 5% bovine serum albumin (BSA) for 1 h. The formation of NETs was detected as described previously (Zhu et al., 2018). Briefly, the cells were incubated with the anti-myeloperoxidase antibody (MPO; ab45977; Abcam, Cambridge, UK), followed by secondary antibody coupled with Alexa Fluor 488 (Thermo Fisher Scientific, Waltham, MA, USA). After removal of the unbound antibodies, DNA was stained using 4′,6-diamidino-2-phenylindole (DAPI). At least two independent observers manually quantified the neutrophils and NETs. The NETs were identified as structures positive for both MPO and DAPI. The images were acquired with an Olympus IX-71 microscope (Olympus, Tokyo, Japan) at magnification of 200×. The percentage of NET-releasing cells was calculated as an average of 10 random fields normalized to the total number of neutrophils.

In order to further confirm the result of immunofluorescence staining, the QuantiFluor dsDNA System (Promega, Madison, WI, USA) was used to quantify the levels of cell-free DNA (cfDNA) in the culture supernatant of neutrophils according to the manufacturer’s instructions. The fluorescence (504 nm Ex/531 nm Em) was measured using a plate reader.

### Measurement of Intracellular ROS

The levels of intracellular ROS were measured using the oxidation-sensitive fluorescent dye H2DCF-DA (20,70-dichlorodihydrofluorescein diacetate, Invitrogen, Carlsbad, CA, USA). Briefly, the cells were loaded with 10 μM H2DCF-DA fluorescence probe, and incubated at room temperature for 30 min in the dark, followed by that in ice bath for 5 min to quench the reaction. Then, the cells were washed one time with PBS, and ROS levels were analyzed immediately by FCM.

### Statistical Analysis

Values were presented as mean ± standard deviation (SD) or mean ± standard error of the mean (SEM), and analyzed using GraphPad Prism 5.01 software. *p*’s were derived from the one-way ANOVA test with *post hoc* analysis and Kruskal-Wallis test. Differences were considered statistically significant at *p* < 0.05.

## Result

### Low-Density Granulocytes in TB Patients Release High Level of Neutrophil Extracellular Traps

**Figure 1:**
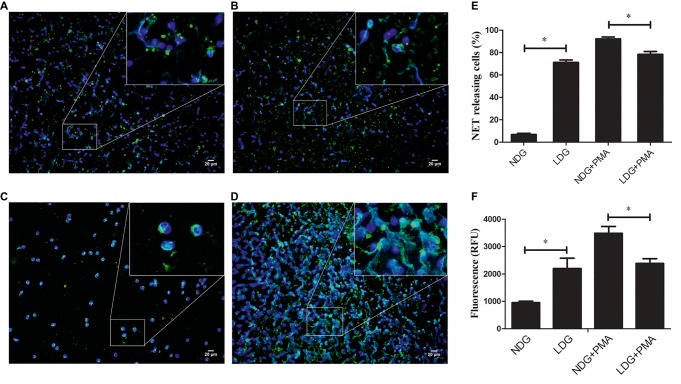
LDGs from tuberculosis patients spontaneously released a high level of NETs. LDGs **(A,B)** and NDGs **(C,D)** were isolated from TB patients and cultured in RPMI 1640 medium containing 3% human serum AB. The cells were either untreated **(A,C)** or stimulated with phorbol 12-myristate 13-acetate (PMA, 20 nM) for 2 h **(B,D)**. MPO was detected using specific antibody (green). Nuclei and extracellular DNA were stained blue with DAPI. The dual-labeled immunofluorescence staining is shown as a merged figure at ×200 as a representative image from 1/5 patients. **(E)** The percentage of NET-releasing cells was calculated manually by at least two independent observers in 10 fields and normalized to the total number of neutrophils, and the results were expressed as mean ± standard error of the mean (SEM) of at least three independent experiments. *p*’s were derived from Kruskal-Wallis test. (F) The levels of cfDNA in the culture supernatant were determined by QuantiFluor dsDNA System, and data were expressed as mean ± standard deviation (SD) of at least three independent experiments. Statistical significance was determined by one-way analysis of variance (ANOVA) followed by *post hoc* test (*p < 0.05).

In a previous study, we found that LDGs in TB patients might originate from *in situ* activation, with increased expression of membrane CD15 and CD66b and decreased expression of membrane CD62L (Deng et al., 2016). Some studies showed that LDGs in patients with autoimmune diseases, such as SLE and RA, spontaneously released high levels of NETs (Zhang et al., 2017; Kanamaru et al., 2018). These studies hinted to us that LDGs in TB patients might also result in high level of NETs. To confirm this hypothesis, LDGs and autologous NDGs in patients with tuberculosis were isolated and cultured in RPMI 1640 medium containing 3% human serum AB. The baseline level of NET was detected using immunofluorescence staining with anti-MPO antibody and DAPI staining. The extracellular NET DNA was qualified by the QuantiFluor dsDNA System. In addition, the levels of NET formation were detected after stimulation with 20 nM PMA. The results showed that LDGs obtained from TB patients spontaneously released a higher level of NETs than autologous NDGs. However, NDGs released a significantly high level of NETs than LDGs after stimulation of PMA (Figure 1).

**Figure 2:**
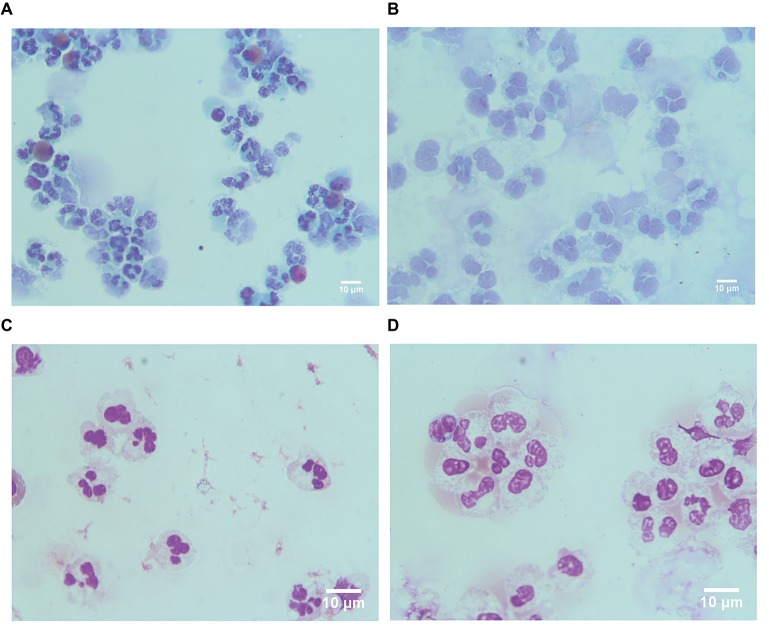
LDGs originated from TB patients show loose nucleus. LDGs **(A,B)** and NDGs **(C,D)** were separated from the TB patient, stained by Papanicolaou stain **(A,C)** and Wright’s stain **(B,D)**, and observed under a microscope (×1,000 magnification).

**Figure 3:**
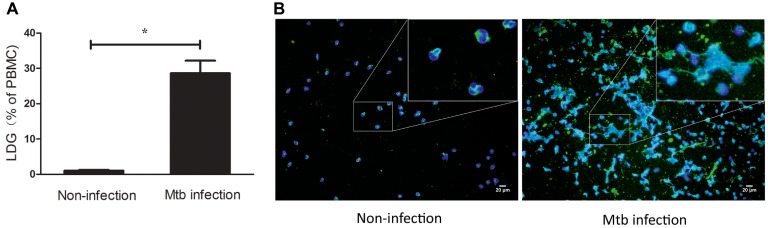
LDGs originated from *in vitro M. tuberculosis* infection released a high level of NETs. Freshly collected whole blood from healthy controls was infected with *M. tuberculosis* for 2 h and centrifuged by Ficoll density gradient centrifugation. LDGs in the PBMCs were identified as CD14^low^ CD15^+^ cells and enumerated by FCM. Data are represented as mean ± SD of at least three independent experiments and the statistical significance was determined by unpaired *t*-test (*p < 0.05) **(A)**. NDGs from the RBC fraction and LDGs from the PBMC fraction were isolated individually by the immunomagnetic negative selective method, stained with anti-MPO (green) antibody and DAPI (blue), and observed under a fluorescence microscope **(B)** (×200).

These results indicated that LDGs in TB patients are primed cells, which spontaneously form a high level of NETs. The morphological observation revealed that the nucleus of LDGs was relatively loose than that in NDGs (Figure 2), which demonstrated chromatin decondensation and release of NETs. Besides, LDGs that originated from peripheral blood of healthy controls by *M. tuberculosis* infection were shown to release a higher level of NETs than those by autologous NDGs (Figure 3).

### Release of Neutrophil Extracellular Traps Is a Mechanism of Low-Density Granulocyte Generation

**Figure 4:**
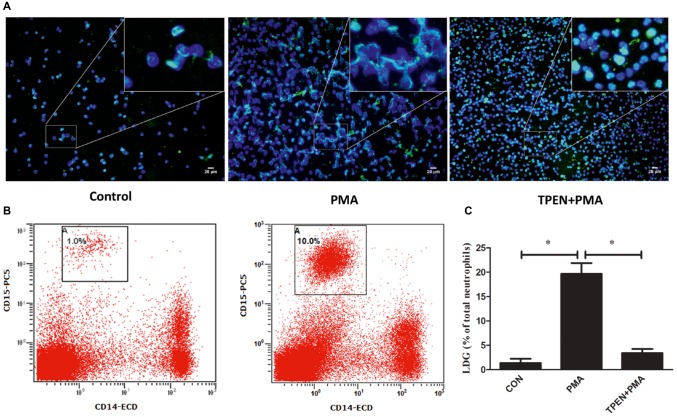
Prompting the release of NETs induces the conversion of NDGs to LDGs. NDGs were separated from healthy controls and pretreated with or without TPEN (10 μM) for 30 min, followed by stimulation with PMA (20 nM) for 2 h. **(A)** The release of NETs was detected by immunofluorescence staining. Nuclei were stained with DAPI (blue), and MPO (green) was detected using specific antibody. The dual-labeled immunofluorescence staining is shown as a merged figure. The magnification was ×200. **(B)** Neutrophils were washed three times, resuspended in 3 ml of sterile saline, and added into 3 ml of freshly collected autologous whole blood. PBMCs in this mixture and another equal division of untreated autologous peripheral blood were immediately isolated by Ficoll density gradient centrifugation. LDGs in these PBMCs were identified as CD14^low^ CD15^+^ cells and enumerated by FCM. **(C)** The number of LDGs in stimulated neutrophils was calculated by subtracting the number of LDGs in the untreated blood with LDGs in the mixture. The results are expressed as mean ± SD of at least three independent experiments, and the statistical significance was determined by one-way ANOVA followed by *post hoc* test (*p < 0.05).

The phenomenon that LDGs purified from both TB patients and those induced from NDGs by *M. tuberculosis* infection could release high levels of NETs prompting the necessity to investigate whether release of NET plays a role in the generation of LDGs. LDGs were purified from TB patients and induced from NDGs by *M. tuberculosis* infection, which released high levels of NETs, thereby necessitating to understand the role of NET release in the generation of LDGs. To explore the putative role of NETs’ release in the generation of LDGs, purified NDGs from healthy controls were pretreated with or without TPEN, a membrane-permeable Zn^2+^-selective chelator, which is essential for NET formation in neutrophils (Hasan et al., 2013) for 30 min, followed by stimulation with NET inducer PMA (Brinkmann et al., 2004) for 2 h. The release of NETs and the conversion of NDGs to LDGs were determined as described in Section “Materials and Methods.” The results showed that LDGs in PBMCs could be distinguished clearly based on the expression of the neutrophil marker CD15 and the monocyte marker CD14, and the conversion of NDGs to LDGs could be calculated with good repeatability (Figure 4B). Additionally, PMA significantly promoted the release of NETs accompanied by the increase in LDG generation. However, when the release of NET was inhibited by TPEN, the generation of LDG was also significantly inhibited upon stimulation with PMA (Figure 4).

**Figure 5:**
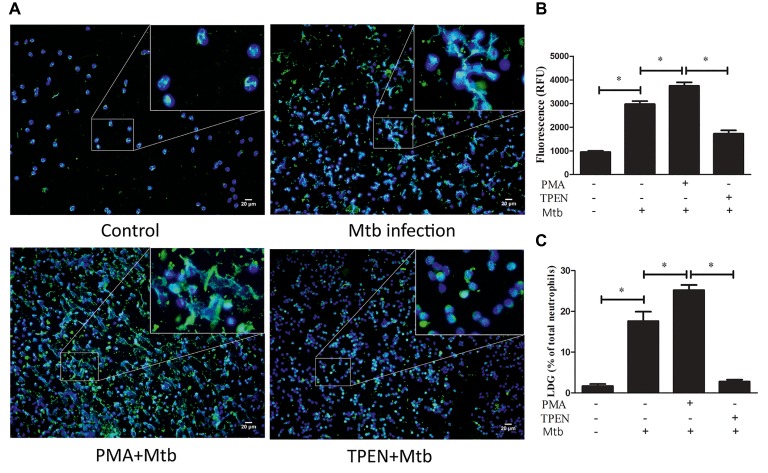
The levels of NET release regulate the conversion of NDGs to LDGs upon *M. tuberculosis* infection. NDGs were isolated from healthy controls, and either untreated or pretreated with TPEN (10 μM) or PMA (20 nM) for 30 min, followed by infection with *M. tuberculosis* (Mtb) for 2 h at the MOI of 5. **(A)** The release of NETs was detected by immunofluorescence staining. Nuclei were stained with DAPI (blue), and MPO (green) was detected with specific antibody. The dual-labeled immunofluorescence staining is shown as a merged figure at ×200. **(B)** The levels of cfDNA in the culture supernatant were determined by QuantiFluor dsDNA System, and results are expressed as mean ± SD of at least three independent experiments. **(C)** Neutrophils were washed three times and resuspended in 3 ml of sterile saline, and then added into 3 ml of freshly collected autologous whole blood. PBMCs in this mixture and a proportion of untreated autologous peripheral blood were immediately isolated by Ficoll density gradient centrifugation. LDGs in these PBMCs were identified as CD14^low^ CD15^+^ cells and counted by FCM. The number of LDGs in these mixtures was calculated by subtracting the number of LDGs in the untreated blood with LDGs in the mixture. The results are expressed as mean ± SD of at least three independent experiments, and the statistical significance was determined by one-way ANOVA followed by *post hoc* test (*p < 0.05).

To further confirm the association between NET release and LDG generation upon *M. tuberculosis* infection, NDGs from healthy controls were untreated or pretreated with TPEN, the inhibitor of NET formation, or PMA, the inducer of NET formation, for 30 min, followed by infection with *M. tuberculosis* at MOI 5. The results showed that PMA further increased the release of NETs in neutrophils and promoted the generation of LDGs post-*M. tuberculosis* infection. Conversely, TPEN significantly inhibited the release of NETs, as well as, the generation of LDGs induced by *M. tuberculosis* infection (Figure 5).

### ROS Are Involved in the Generation of Low-Density Granulocytes

**Figure 6:**
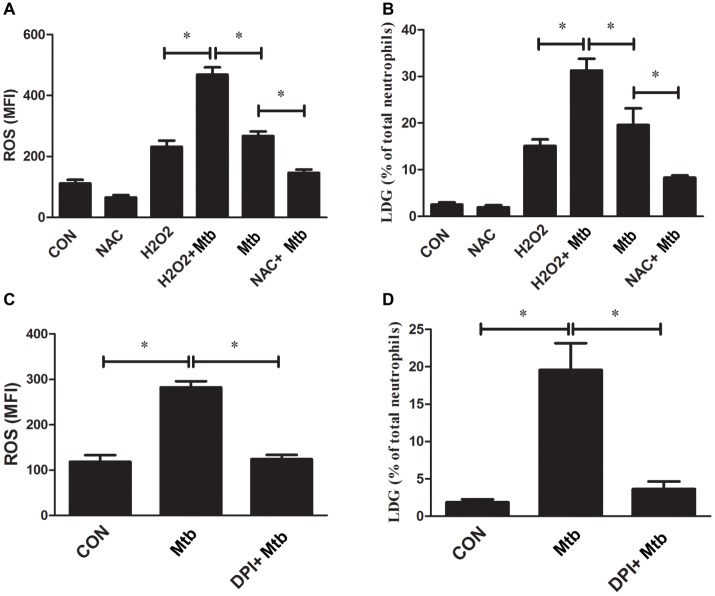
The level of ROS regulates the generation of LDG. NDGs were isolated from healthy controls and either untreated or pretreated with NAC (30 μM) or H_2_O_2_ (50 μM) for 30 min, and then, infected with or without *M. tuberculosis* for 2 h. **(A)** Cells were loaded with H2DCF-DA fluorescence probe (10 μM), and the intracellular ROS levels were determined by FCM. **(B)** The generation of LDGs was detected as described above. **(C,D)** NDGs were separated from healthy controls and pretreated with or without DPI (25 μM) for 30 min, followed by infection with *M. tuberculosis* for 2 h. The intracellular ROS levels in neutrophils and generation of LDNs were detected as described above. The results are expressed as mean ± SD of at least three independent experiments and the statistical significance was determined by one-way ANOVA and *post hoc* test (*p < 0.05).

The above results demonstrated that NETs’ release is one of the mechanisms of *M. tuberculosis* infection-related LDG generation. Previous studies have proved that the formation of NETs is dependent on the production of ROS (Fuchs et al., 2007; Papayannopoulos et al., 2010). Our previous study also found that LDGs from TB patients produced increased levels of ROS than autologous NDGs (Deng et al., 2016). These studies suggested that ROS might be involved in the generation of TB-related LDGs. To confirm this hypothesis, purified NDGs from healthy controls were infected with or without *M. tuberculosis*, and the intracellular ROS levels were detected. The results showed that the *in vitro* infection with *M. tuberculosis* significantly increased the levels of intracellular ROS and promoted the conversion of NDGs to LDGs (Figures 6A,B). In combination with our previous findings (Deng et al., 2016), the present results showed that both *in vitro* and *in vivo* infection of *M. tuberculosis* significantly increased the intracellular levels of ROS in neutrophils and the frequency of LDGs.

Next, purified NDGs from healthy controls were pretreated with the ROS scavenger NAC or oxidizer H_2_O_2_ for 30 min, followed by infection with *M. tuberculosis* for 2 h and detection of intracellular ROS levels and generation of LDGs. The results confirmed that the formation of NETs was associated with the levels of ROS (data not shown), and showed that H_2_O_2_ significantly increased the levels of intracellular ROS in neutrophils accompanied by the increased level of LDG generation even without an infection. Also, H_2_O_2_ further increased the level of LDG generation under *M. tuberculosis* infection. However, NAC pretreatment significantly decreased the ROS levels in *M. tuberculosis*-infected NDGs and suppressed the conversion of NDGs to LDGs upon *M. tuberculosis* infection (Figures 6A,B).

To further confirm the association between ROS and *M. tuberculosis* infection-related LDGs, purified NDGs from healthy controls were untreated or pretreated for 30 min with DPI, an inhibitor of neutrophil NADPH oxidase, and then infected with *M. tuberculosis*. The generation of LDGs was detected at 2 h post-infection. Data showed that DPI suppressed the generation of intracellular ROS in neutrophils and inhibited the generation of *M. tuberculosis-*induced LDGs (Figures 6C,D).

### ROS Induce the Generation of Low-Density Granulocytes by Regulating the Formation of Neutrophil Extracellular Traps

**Figure 7:**
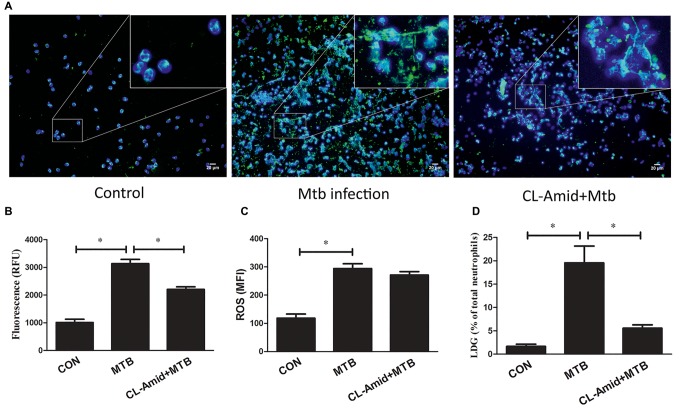
NDGs were separated from healthy controls, those either untreated or pretreated with Cl-amidine (200 μM) for 30 min, and infected with *M. tuberculosis* for 2 h at the MOI of 5. **(A)** The formation of NETs was detected by dual-labeled immunofluorescence. Nuclei were stained with DAPI (blue), and MPO (green) was detected using specific antibody. **(B)** The levels of cfDNA in the culture supernatant were determined by QuantiFluor dsDNA System. **(C)** The intracellular ROS levels were determined by FCM after the loading of H2DCF-DA fluorescence probe (10 μM). **(D)** The generation of LDGs was detected as described above. The results are expressed as mean ± SD of at least three independent experiments, and the statistical significance was determined by one-way ANOVA, followed by *post hoc* test. *p < 0.05. CON, group received no treatment; Mtb, *M. tuberculosis* infection.

The results indicated that the increased ROS level promotes the formation of NETs and the generation of LDGs, while the inhibition of ROS production can decrease the release of NETs and the generation of LDGs after *M. tuberculosis* infection. These observations demonstrated that ROS could promote the generation of *M. tuberculosis*-related LDGs, probably by regulating the formation of NET. To verify this hypothesis, Cl-amidine, an inhibitor of PAD4 which was commonly used as the chromatin decondensation and NET formation but did not affect the levels of intracellular ROS, was used to pretreat the purified NDGs from healthy controls for 30 min before *M. tuberculosis* infection. The results showed that Cl-amidine significantly inhibited the release of NETs after the infection. Although *M. tuberculosis* infection induced similar levels of intracellular ROS in neutrophils of both groups, fewer neutrophils that received Cl-amidine pretreatment were converted to LDGs as compared to those that did not receive pretreatment (Figure 7).

## Discussion

The roles and characteristics of LDG are currently under intensive focus in the fields of cancer, autoimmune diseases, and infectious diseases, wherein, the fluctuation in the LDG proportion presents a correlation with the severity of the disease and responsiveness to the treatment (Cloke et al., 2012; Carmona-Rivera and Kaplan, 2013; Sagiv et al., 2015; Deng et al., 2016). The variety in immunophenotype and specific functions in different diseases makes the LDGs be categorized into two main subgroups, immunosuppressive and pro-inflammatory. In some diseases such as solid tumors and hematological malignancies, LDGs were also named as granulocyte-like myeloid-derived suppressor cells (G-MDSCs) (Sagiv et al., 2015; Moses and Brandau, 2016). While in other conditions such as anti-neutrophil cytoplasmic antibody-associated vasculitis and chronic granulomatous disease, LDGs are mainly characterized as pro-inflammatory cells that mediate enhanced proinflammatory and cytotoxic responses as compared to those of autologous NDGs (Denny et al., 2010; Grayson et al., 2015). Our previous study revealed that LDGs in patients with tuberculosis are activated proinflammatory cells with mature neutrophils phenotype (Deng et al., 2016).

Recent studies suggested that LDGs might be involved in the occurrence and development of some diseases and may be a promising target for the treatment (Grayson et al., 2015; Rocha et al., 2015; Liu et al., 2017). Thus, the origin of LDGs in different conditions has also attracted increasing attention. A study based on the cytogenetic microarray analysis demonstrated that LDGs in SLE patients are a kind of immature neutrophil that result from disruption of granulocyte development (Singh et al., 2014). However, another study in SLE patient proposed that LDGs arise as a consequence of *in situ* activation of normal neutrophils (Garcia-Romo et al., 2011), which was confirmed subsequently in a murine model of cancer, wherein NDGs exposed to transforming growth factor beta 1 (TGF-β1) were switched into LDGs (Sagiv et al., 2015). Our recent study also proved that the *in situ* activation contributes to the generation of LDGs in *M. tuberculosis* infection (Deng et al., 2016).

Nonetheless, the molecular mechanism underlying the generation of LDGs is yet to be elucidated. We found that LDGs in patients with tuberculosis is a population of activated neutrophils that originated from *in situ* activation. The present study demonstrated that LDGs from TB patients spontaneously released a higher level of NETs than that by autologous NDGs, but released fewer NETs than the autologous NDGs after PMA stimulation. This phenomenon indicated that LDGs in TB patients are primed cells, which spontaneously form high level of NETs. These results are in agreement with the previous reports in other disease models (Denny et al., 2010; Fu et al., 2014; Zhang et al., 2017).

NETs are complex structures consisting of extracellular chromatin decorated with granular and cytoplasmic proteins, such as MPO and neutrophil elastase (Brinkmann et al., 2004). NETs have been described as a beneficial mechanism of host defense against pathogens that can immobilize and kill the pathogens. However, recent studies suggested that NET may be detrimental in various diseases such as cancer and atherosclerosis (Brinkmann et al., 2004; Nauseef, 2007; Kaplan and Radic, 2012). Consistently, NETs have also been shown to be released by *M. tuberculosis*-infected neutrophils. And, it was found that NETs could not kill *M. tuberculosis*, but was closely related to tuberculosis-related lung tissue damage (Ramos-Kichik et al., 2009; de Melo et al., 2019). The release of NETs is the result of several successive events. First, stimuli such as PMA, LPS, and bacteria activate the neutrophils. Then, the nuclear chromatin decondenses and the nuclear envelope disintegrates, followed by the release of nuclear content into the cytoplasm, and the nuclear chromatin, cytoplasmic, and granular components are mixed. Ultimately, the cell membrane ruptures and the mixture is released into the extracellular space by a process that is distinct from necrosis or apoptosis (Brinkmann et al., 2004; Yang et al., 2016).

PMA is the most common inducer for the formation of NETs (Brinkmann et al., 2004; Fuchs et al., 2007). In order to reveal whether the release of NETs plays a role in the generation of LDGs, purified NDGs were treated with PMA to trigger the release of NETs, and the conversion of NDGs to LDGs after *M. tuberculosis* infection was determined. The results showed that PMA significantly promoted the release of NETs accompanied by the increase in the generation of LDGs. In addition, the pretreatment with PMA further increases the level of LDGs post-*M. tuberculosis* infection, indicating that the NET release can induce the conversion of NDGs to LDGs. Furthermore, TPEN, a membrane-permeable Zn^2+^-selective chelator and inhibitor of NET formation (Hasan et al., 2013), blocks the conversion of NDGs to LDGs induced by *M. tuberculosis* infection. These results proposed that the release of NETs is one of the mechanisms involved in the generation of LDGs in *M. tuberculosis* infection. Considering that NETs’ release is associated with activation and tissue damage of tuberculosis (Schechter et al., 2017; de Melo et al., 2019), this study suggests that the generation of LDGs may also be associated with the activation of tuberculosis.

The process of NET formation is dependent on the generation of ROS by NADPH oxidase (Kohchi et al., 2009), and the stimulation with both PMA and *M. tuberculosis* triggers the activation of NADPH oxidase (Fuchs et al., 2007). Our previous study also found that LDGs from patients with tuberculosis produce significantly high levels of ROS than autologous NDGs (Deng et al., 2016). Therefore, we assessed whether ROS are involved in the generation of LDGs. The results showed that *M. tuberculosis* triggered the production of ROS and the generation of LDGs. Furthermore, H_2_O_2_ also promoted the generation of LDGs. The pretreatment with NAC, an antioxidant which scavenges the hydroxyl and superoxide radicals (Ahmed et al., 2011; Dean et al., 2011), significantly decreases the levels of intracellular ROS, inhibits the production of NETs, and decreases the generation of LDGs post-*M. tuberculosis* infection. Also, the NADPH oxidase inhibitor DPI blocks the release of NETs and prevents the conversion of NDGs to LDGs after *M. tuberculosis* infection. The current results revealed that the levels of intracellular ROS play a major role in regulating the production of NETs and generation of LDGs in *M. tuberculosis* infection.

Next, we evaluated whether ROS is required for the generation of LDGs in *M. tuberculosis* infection. Since NET formation is ROS-dependent, the release of NETs while decreasing or maintaining the levels of ROS cannot be triggered. Thus, we adopted an alternative strategy to investigate the correlation among ROS levels, NET formation, and LDG generation. Cl-amidine, a highly specific peptidylarginine deiminase 4 (PAD4) inhibitor (Knight et al., 2013) was used in this study to inhibit the formation of NETs without altering the levels of intracellular ROS. PAD4 catalyzes the citrullination of proteins, including histone, which is an obligatory step preceding chromatin decondensation and release, thereby exerting a crucial role in NET formation (Leshner et al., 2012; Rohrbach et al., 2012). Previous studies and current results showed that Cl-amidine significantly inhibits the formation of NETs in human neutrophils *in vitro* and mouse neutrophils *in vivo* (Leshner et al., 2012; Rohrbach et al., 2012), but does not alter the intracellular ROS levels in neutrophils (Knight et al., 2014). Although the ROS levels were robustly increased after *M. tuberculosis* infection, however, Cl-amidine could prevent the conversion of NDGs to LDGs after *M. tuberculosis* infection, which suggested that ROS-induced generation of LDGs is mediated by regulating the formation of NETs in neutrophils.

In summary, the present and our previous studies demonstrate that the infection of *M. tuberculosis* can induce the conversion of NDGs to LDGs, and the generation of *M. tuberculosis* infection-related LDGs is related to the intracellular ROS level and NET release in neutrophils. To the best of our knowledge, the molecular mechanisms underlying the generation of LDGs has not yet been reported. Thus, the present study, for the first time, reveals that ROS contributes to the generation of LDGs by regulating the formation of NETs in *M. tuberculosis* infection.

## Ethics Statement

This study was approved by the Ethical Review Committees of the First Affiliated Hospital of Nanchang University and was carried out in compliance with the Helsinki Declaration. Written informed consent was obtained from all the subjects enrolled in this study.

## Author Contributions

JL conceived the project and wrote the manuscript. JL, RS, and YP designed the experiments, collected the study subjects, performed the experiments, and analyzed the data. RS, YP, ZD, YD, JY, YG, ZH, QL, and HJ performed the experiments.

### Conflict of Interest Statement

The authors declare that the research was conducted in the absence of any commercial or financial relationships that could be construed as a potential conflict of interest.
